# Control-FREEC viewer: a tool for the visualization and exploration of copy number variation data

**DOI:** 10.1186/s12859-024-05694-w

**Published:** 2024-02-14

**Authors:** Valentina Crippa, Emanuela Fina, Daniele Ramazzotti, Rocco Piazza

**Affiliations:** 1 Department of Medicine and Surgery, University of Milano-Bicocca, Monza, Italy; 2 Department of Thoracic Surgery, IRCCS San Raffaele Scientific Institute, Milan, Italy; 3 Fondazione IRCCS San Gerardo Dei Tintori, Monza, Italy

**Keywords:** Copy number variation, Data visualization, Data exploration

## Abstract

**Background:**

Copy number alterations (CNAs) are genetic changes commonly found in cancer that involve different regions of the genome and impact cancer progression by affecting gene expression and genomic stability. Computational techniques can analyze copy number data obtained from high-throughput sequencing platforms, and various tools visualize and analyze CNAs in cancer genomes, providing insights into genetic mechanisms driving cancer development and progression. However, tools for visualizing copy number data in cancer research have some limitations. In fact, they can be complex to use and require expertise in bioinformatics or computational biology. While copy number data analysis and visualization provide insights into cancer biology, interpreting results can be challenging, and there may be multiple explanations for observed patterns of copy number alterations.

**Results:**

We created Control-FREEC Viewer, a tool that facilitates effective visualization and exploration of copy number data. With Control-FREEC Viewer, experimental data can be easily loaded by the user. After choosing the reference genome, copy number data are displayed in whole genome or single chromosome view. Gain or loss on a specific gene can be found and visualized on each chromosome. Analysis parameters for subsequent sessions can be stored and images can be exported in raster and vector formats.

**Conclusions:**

Control-FREEC Viewer enables users to import and visualize data analyzed by the Control-FREEC tool, as well as by other tools sharing a similar tabular output, providing a comprehensive and intuitive graphical user interface for data visualization.

## Background

Copy number alterations (CNAs) are genetic variations that cause an abnormal increase or decrease in the number of copies of a genomic region, and they are commonly detected in cancer. CNAs can affect various regions of the genome, including broad regions that encompass multiple genes, individual genes, or even non-coding RNA molecules of small size. CNAs contribute to tumorigenesis and can have a significant impact on the progression of cancer, by influencing the level of gene expression, disturbing regulatory networks, and compromising genomic stability. In cancer research, copy number data analysis employs computational techniques to detect and scrutinize CNAs from genomic data obtained via high-throughput sequencing platforms like whole-genome sequencing, whole-exome sequencing, and array-based technologies. The primary objective of copy number data analysis is to pinpoint frequently occurring CNAs and comprehend their functional implications in the context of cancer biology [1, 2].

Various techniques can be employed to detect CNAs, including segmentation-based algorithms, which divide the genome into distinct segments based on copy number patterns, and breakpoint-based algorithms, which determine the precise location of copy number variations. After identifying CNAs, subsequent analyses can encompass gene set enrichment analysis, pathway analysis, and functional annotation to unravel the biological implications of the modifications. Copy number data analysis has emerged as a crucial aspect of cancer research since it offers a glimpse into the fundamental genetic mechanisms that trigger cancer growth and advancement. These insights can potentially pave the way for identifying innovative therapeutic targets and devising more efficient treatments for cancer patients.

In cancer research, several tools are available for visualizing copy number data [3], such as Nexus Copy Number (BioDiscovery), IGV [4], cBioPortal [5, 6], and the UCSC Genome Browser [7]. These tools offer valuable capabilities for exploring and analyzing copy number variations within the context of genomic annotations and reference sequences, providing insights into the underlying genetic mechanisms that drive cancer development and progression. Features such as heatmaps, boxplots, scatterplots, and track hubs enable researchers to identify recurrent CNAs, visualize chromosomal aberrations, and compare copy number data across various cancer types and subtypes. However, some of these tools can be complex and demand expertise in bioinformatics or computational biology for effective utilization.

Despite the availability of various CNA detection tools in the literature, there are still limitations in CNA viewers. CNV-ClinViewer [8], for instance, provides a user-friendly Web application focused on clinical CNA annotations and interpretation, using genomic coordinates of CNAs from human reference genomes GRCh37/hg19 or GRCh38/hg38 as input. However, this feature limits researchers working with different organisms, like mice or drosophila, as there's no option to upload alternative reference genomes.

Other visualization tools like aCNViewer [9] can offer genome-wide visualization of chromosomal aberrations for sample groups, providing three different graphical representations. Meanwhile, CNView [10] is designed for visualization, statistical scoring, and annotations of CNAs in whole-genome sequencing datasets. But both these tools have the limitation that they require R for access, which may not be as user-friendly as web-based interfaces.

All in all, while tools for visualizing copy number data in cancer research offer valuable capabilities, they also have limitations. Some tools for visualizing copy number data can be complex and require expertise in bioinformatics or computational biology to be used effectively. Moreover, while copy number data analysis and visualization can provide valuable insights into cancer biology, interpreting the results can be challenging, and there may be multiple explanations for the observed patterns of CNAs.

To address this gap, we developed Control-FREEC Viewer, a tool for effectively visualizing and exploring copy number data. Control-FREEC Viewer allows users to import copy number data analyzed by the Control-FREEC tool or by tools sharing a similar tabular output and provides a comprehensive and intuitive graphical user interface for visualizing the data.

## Implementation

ControlFREECViewer is entirely written in C# using the.NET Framework v.4.7.2 and implements an event-driven architecture, to react to user-driven events and act on them in real time, targeting 64bit platforms. Copy number visualization plots are built using the *DataVisualization* class, runtime version v.4.0. ControlFREECViewer accepts as input *bam_ratio* files, which are the standard, tab-separated output files of copy number analysis tools such as ControlFREEC and contain the following tab-separated columns: ‘Chromosome’, ‘Start’, ‘Ratio’, ‘MedianRatio’, ‘CopyNumber’.

Together with the *bam_ratio* file, ControlFREECViewer requires two annotation files: 1) a Gene transfer format (GTF) file, which holds information about gene structure. ControlFREECViewer uses it to extract the coordinates of all the genes and exons to build the reference genome and to integrate gene/exons coordinates with copy number window data reported in the *bam_ratio* input file in the main ControlFREECViewer window. 2) A cytoBand annotation file, which is a five-column tab-delimited text file describing the position of all the cytogenetic bands of the target genome. The cytoband file can be directly downloaded from the UCSC Genome Browser as a "cytoBandIdeo.txt.gz" file from the Mapping and Sequencing—> Chromosome Band (Ideogram)—> cytoBandIdeo Table Browser. ControlFREECViewer uses the cytoband data to generate the chromosome plot and to calculate the size of each chromosome.

ControlFREECViewer comes with four gtf/cytoBand reference pairs directly available: human hg38, human hg19, mouse mm39 and mouse mm10.

The main ControlFREECViewer classes are represented as follows. The *GenomeCopyNumber* class accepts as input a *bam_ratio* path and a hashset containing all the valid chromosomes extracted by the input gtf file and implements all the logic required to read the *bam_ratio* using an internal stream. All the chromosomes that are not present in the hashset are discarded. Information pertaining the copy number values of all the *bam_ratio* windows are internally stored in a dictionary, whose key is represented by chromosome names and values by an ordered list of *WindowCopyNumber* objects.

The *WindowCopyNumber* class stores all the information pertaining each window, specifically the start position (32-bit integer), the Log_2_ window copy number ratio (32 bit double), the median Log_2_ copy number ratio for the whole segment (32 bit double) and the predicted segment allele count (32-bit integer).

The *GenesInfo* class accepts as input a gtf file, either gzipped or uncompressed, and stores gene information in a dictionary, whose key is represented by chromosome names and value by an ordered list of *Gene* objects.

The *Gene* class stores information pertaining individual genes, among them chromosome and Gene name (string), gene start and end (32-bit integers), an ordered list of exon start and end positions (List < Int32 > objects).

The *CytoBand* class accepts as input a cytoband path plus an ordered list of valid chromosomes (as a List < string > object). By reading the cytoband data using an internal *streamreader* object, *CytoBand* generates a set of 3 main objects:

1. A Dictionary < string, List < CytoBandSegment >  > which stores the chromosome name as key (string) and an ordered list of *CytoBandSegment* objects as values.
2. The *CytoBandSegment* class stores the coordinates (32-bit integer), name (string) and Giemsa stain color (24-bit RGB Color) of a cytoband segment.
3. A list of tuples of type < string, integer > containing the name and associated size in bases of all the valid chromosomes, as derived from the *GenesInfo* object.
4. A Dictionary of type < GiemsaStain, Color > , used to map chromosomal cytoband types to an associated, constant 24-bit RGB color. The *GiemsaStain* is an enumeration class representing the following Giemsa staining constant items: *gneg*, *gpos25*, *gpos50*, *gpos75*, *gpos100*, *acen*, *gvar* and *stalk*.

## Results

### Loading an experiment

**Fig. 1:**
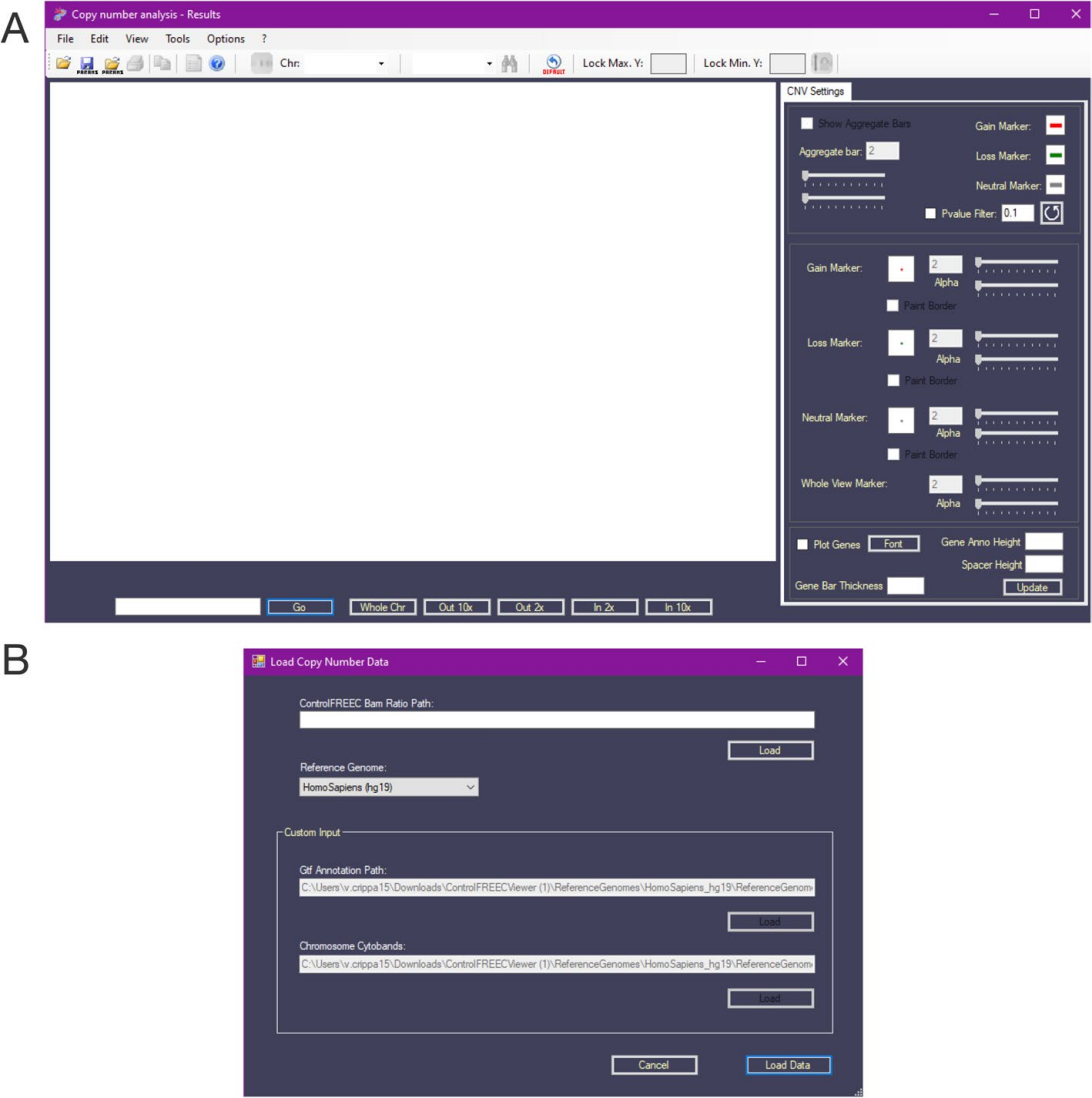
Loading an experiment. **A** The main Control-FREEC Viewer screen. **B** The Load Data form. This form enables users to load data generated by Control-FREEC in the form of a tabular.txt file

The Control-FREEC Viewer is a tool designed for visualizing and exploring copy number variation data. It can process input data in the format generated by Control-FREEC [11], whose resulting data can be loaded as a flat, tabular.txt file. To visualize the results, the user can load an experiment by either clicking on the folder icon located at the top left of the screen or by navigating to the 'File' menu and selecting 'Open File', and then loading the file generated by Control-FREEC (ControlFREEC Bam Ratio Path) and the reference exome for the desired species such as human, mouse, or other custom reference genomes (see Fig. 1).

### Data visualization: whole genome view

**Fig. 2:**
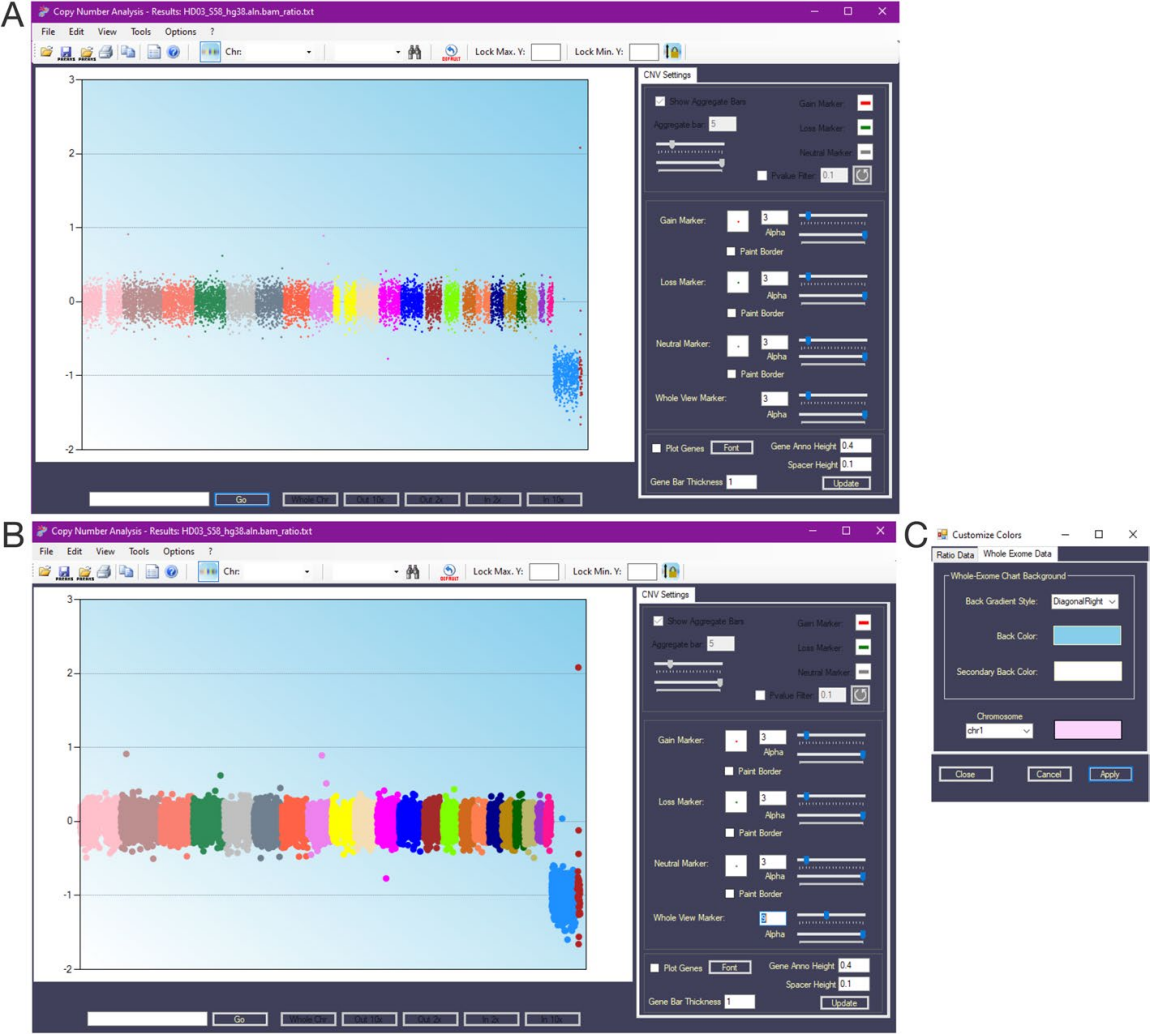
Data visualization. **A** The Whole Genome View of the copy number of the 23 chromosomes, represented with different colors. **B** The same visualization of **A** with different dimensions of the Whole View Marker. **C** The Customize Colors panel

Once the data from Control-FREEC and the reference exome are loaded, the software will visualize the chromosomes in different colors in the *Whole Genome View*. The copy numbers are reported as log2 ratios relative to the normal ploidy of 2, that is ${{\text{log}}}_{2}\frac{Copy\, number}{2}$. The example shown in Fig. 2A displays the 22 autosomes with an average of 2 copies, which results in a distribution of values near 0, as expected. In contrast, the sex chromosomes are represented with a single copy, and therefore, show a negative value on the y-axis (y = -1). The right panel shown in Fig. 2A (*CNV Settings*) allows users to modify the graphic visualization of the *Whole Genome View*. Specifically, users can adjust the size and transparency (*alpha*) of the displayed markers by using the sliders associated with the *Whole View Marker* parameters. As an example, Fig. 2B shows the results of modifying the *Whole View Marker* from 3 to 9. Additionally, users can customize the colors of the *Ratio Chart Background* and the *Whole-Exome Chart Background* by clicking on 'Options', selecting 'Set Colors', and applying the new configuration, as shown in Fig. 2C.

### Data visualization: single chromosome view

**Fig. 3:**
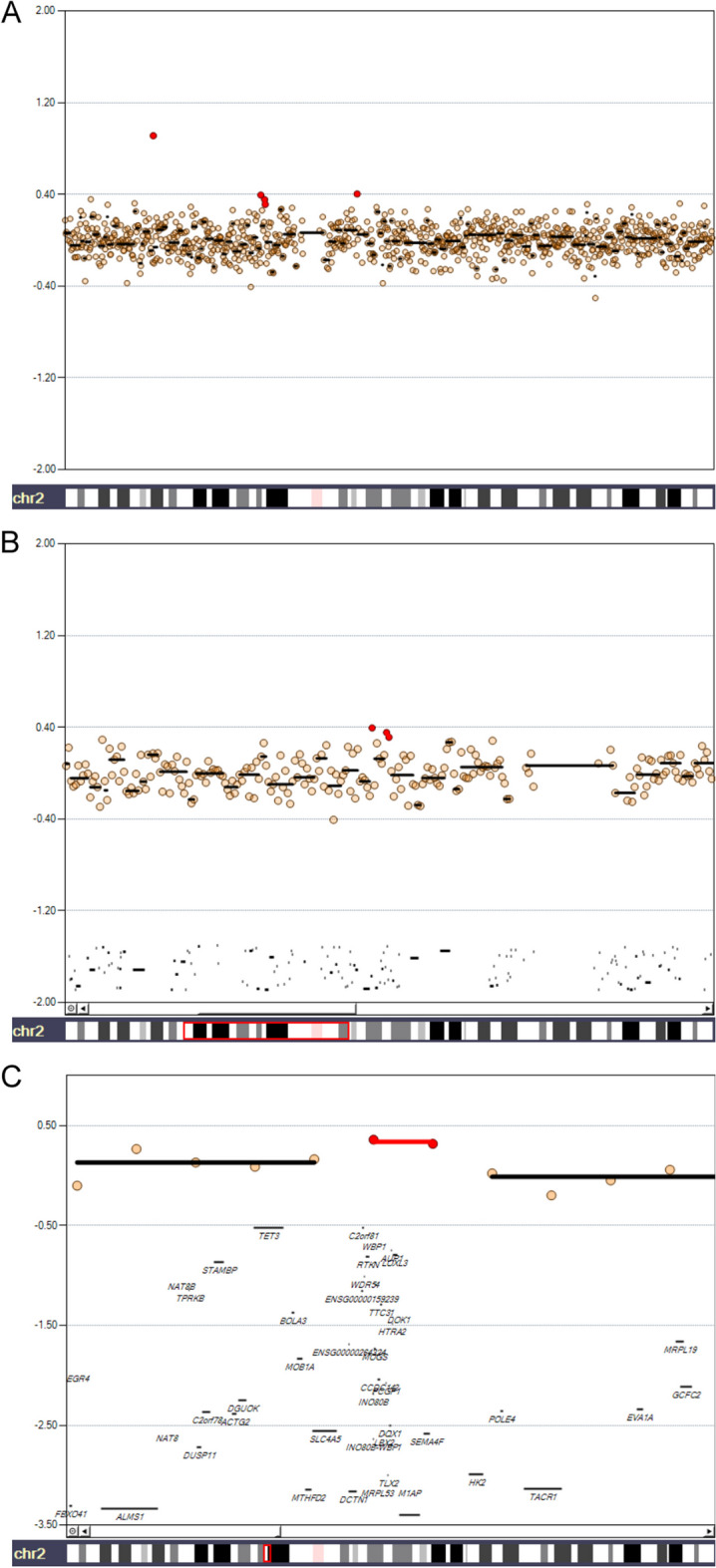
Single-Chromosome View of chromosome 2 with visual preset Publication- > Warm, with gain markers in red and neutral markers in orange. **A** Whole chromosome 2 view. **B** Zoom in pericentromeric region of chromosome 2. Thick, black lines in the lower part of the plot represent annotated genes. The red box in the bottom highlights the chromosome region displayed in the plot. **C** Further zoom-in over the amplified region, highlighted by the two red circles and the red bar. In the bottom part of the figure, detailed gene annotations are shown

Clicking on a single chromosome allows users to zoom in and explore the gain and loss markers for that specific chromosome, which are represented in different colors compared to the neutral markers. In this example, the gain markers are colored in red (Fig. 3A). Users can modify the *Single Chromosome View* using the panel on the right. For both the aggregation bar and the gain, loss, and neutral markers, users can adjust the size, color, and transparency. The bottom panel provides four buttons that allow users to zoom in (2X and 10X) or zoom out on a specific region of the chromosome. When users perform a zoom-in, the specific zoomed region is highlighted on the chromosome (Fig. 3B). Figure 3C shows a further zoom-in over the amplified region of chromosome 2, with detailed genes annotated on the bottom.

### Finding copy number gain or loss on a specific gene

**Fig. 4:**
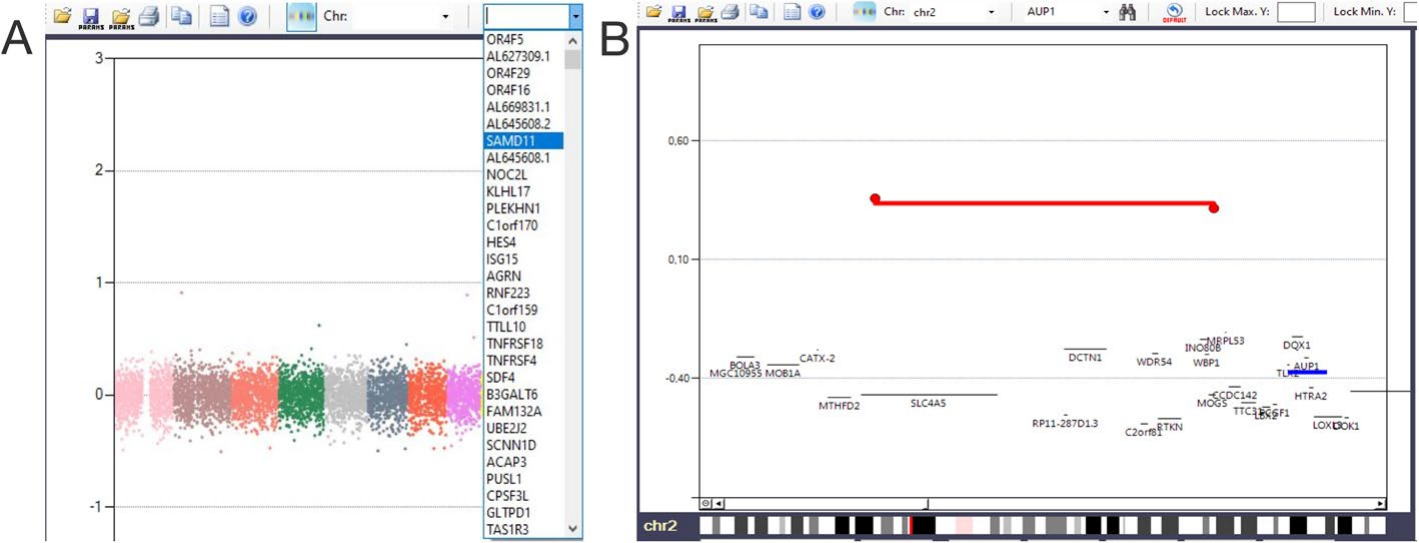
Finding copy number variation of a gene through its name. **A** Whole Genome View; on the background chromosomes are represented using different colors. A drop-down menu allows the users to select the gene of interest. **B** AUP1 gene is found using the gene-finding function, as shown in the upper section of the panel. The blue line highlights the position of the AUP1 gene

To visualize the copy number gain or loss of a specific gene, users can select the gene of interest from the drop-down menu and click on the binoculars icon on the right (Fig. 4). Figure 4B shows an example of the copy number variation visualization for the *AUP1* gene on chromosome 2.

### Saving a figure and the analysis parameters

**Fig. 5:**
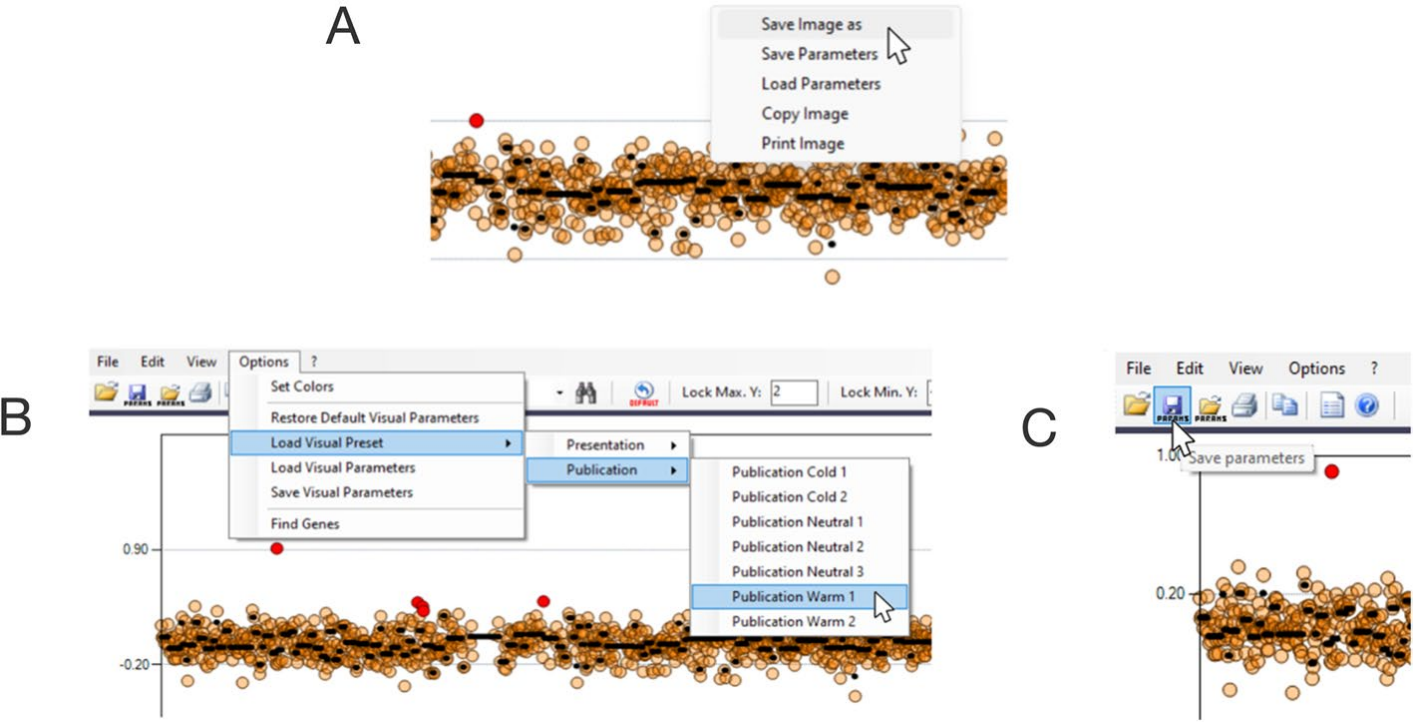
Saving a figure and the analysis parameters. **A** Representative examples of how to save an image. **B**, **C** Load Visual Preset and save analysis parameters

To save a specific figure, users need to click on *File*, *Save Image* (Fig. 5A), choosing both raster as well as vector image formats. Users can modify the picture background for publication or presentation by clicking on *Load Visual Preset*, *Presentation/Publication*, and then selecting their preferred representation (Fig. 5B). To save the analysis parameters, such as point color and size, transparence, background color, chromosome colors, aggregate lines color and thickness, for future analysis, users can click on the floppy disk icon on the left (Fig. 5C).

### Example of analysis of known copy-number events

**Fig. 6:**
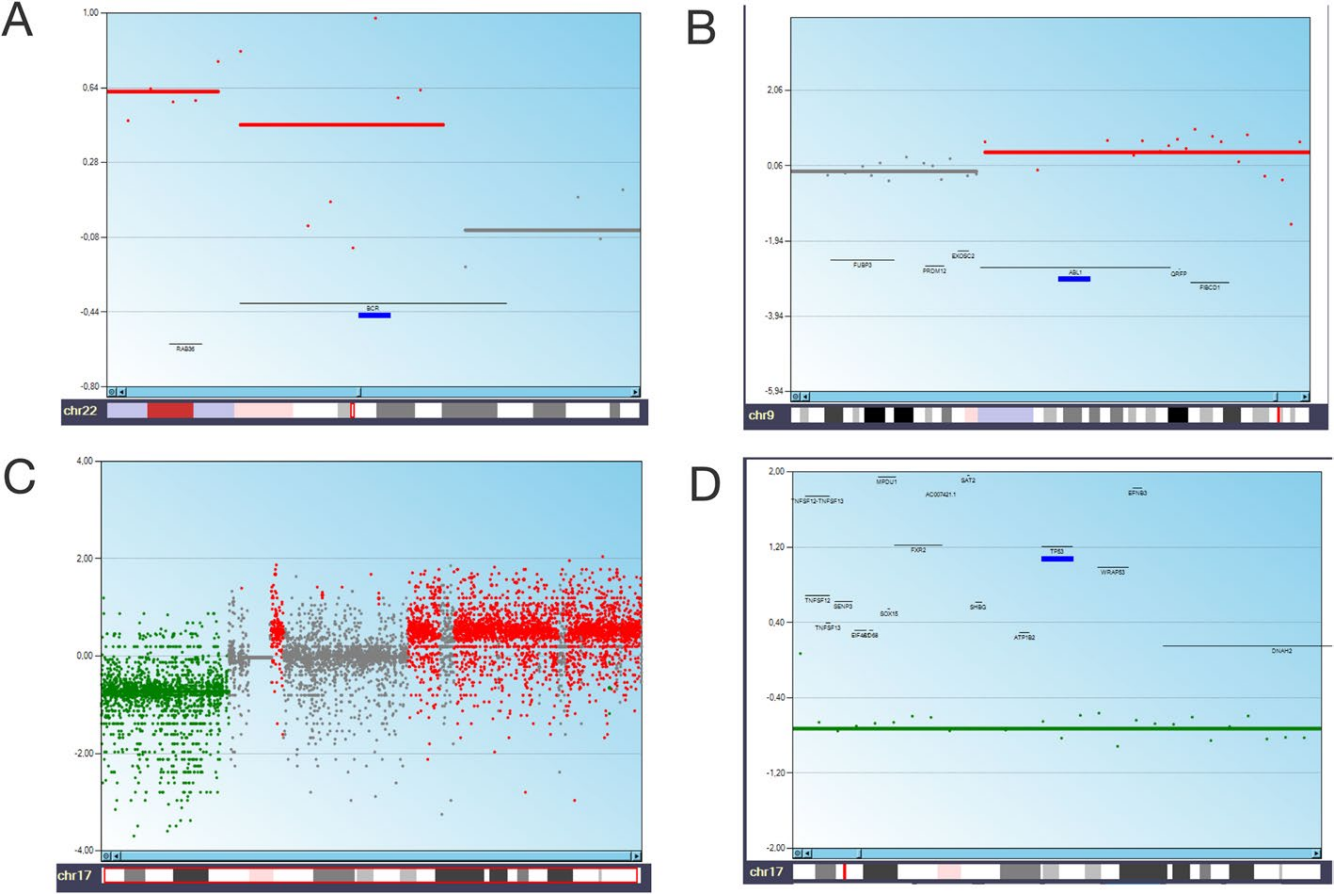
Analysis of known copy-number events of CML patients in blast crisis: CML002BC (**A**, **B**) and CML004BC (**C**, **D**). Copy number gain region on chromosome 22 (**A**) and on chromosome 9 (**B**) is indicative of the amplification of *BCR::ABL1* gene. (**C**) Copy number losses (in green) and gains (in red) at chromosome 17 level. (**D**). Loss of *TP53* at chromosome 17. The blue line highlights the position of the* BCR*, *ABL1* and *TP53 *genes

We provide whole-exome copy number data obtained from two Chronic Myeloid Leukemia (CML) patients (CML002 and CML004) in advanced blast crisis (CML002BC and CML004BC) vs chronic phase (CML002CP and CML004CP) to identify the anomalies associated with disease progression [12]. In particular, the comparison between CML002BC and CML002CP is interesting because upon progression it shows the occurrence of a copy number gain region on chromosome 22 (Fig. 6A) and on chromosome 9 (Fig. 6B), which is the result of *BCR::ABL1* fusion amplification occurring in the t(9;22) chromosome also known as the ‘Philadelphia chromosome’. In CML004, on the other hand, upon progression we observe a deletion of the entire chr7, and at the chr17 level, a heterogeneous situation with both losses and gains. Specifically, we see loss of 17p, resulting in the loss of *TP53* (Fig. 6C, D).

## Conclusions

Various tools are available for visualizing copy number data in cancer research, however many of them offer very limited customization, fail to generate publication quality images, only provide static plots, greatly limiting the ability to explore the data, or require a significant level of bioinformatics or computational biology expertise to use effectively. Additionally, interpreting results from copy number data analysis and visualization can be difficult, as there may be multiple explanations for observed patterns of copy number alterations.

To address these limitations, the Control-FREEC Viewer tool was developed to support researchers visualize and explore copy number data more efficiently. Our framework allows users to import data that has already been analyzed by the Control-FREEC tool, which is then presented using an intuitive graphical user interface. Our software enables users to visualize the data in a comprehensive manner, which can lead to a more in-depth understanding of copy number variations and their role in cancer biology. Overall, the Control-FREEC Viewer tool provides a valuable resource to researchers in the task of enhancing their understanding of copy number alterations in cancer.

## Data Availability

Software is provided as a self-contained application, requiring no installation to be run. Project name: Control-FREEC Viewer. Project home page: https://osf.io/uhs3q/?view_only=30d62e9ebf7949efb8fb07bfb700ab59. OperAting system(s): Microsoft Windows. Programming language: C#. License: Apache License 2.0. Any restrictions to use by non-academics: Apache License 2.0.

## Abbreviations

CNAs: Copy number alterations
GTF: Gene transfer format
CML: Chronic myeloid leukemia
